# A Shared Nephroprotective Mechanism for Renin-Angiotensin-System Inhibitors, Sodium-Glucose Co-Transporter 2 Inhibitors, and Vasopressin Receptor Antagonists: Immunology Meets Hemodynamics

**DOI:** 10.3390/ijms23073915

**Published:** 2022-04-01

**Authors:** Giovanna Capolongo, Giovambattista Capasso, Davide Viggiano

**Affiliations:** 1 Department of Translational Medical Sciences, University of Campania “L. Vanvitelli”, 80138 Naples, Italy; giovanna.capolongo@unicampania.it (G.C.); gb.capasso@unicampania.it (G.C.); 2 BioGeM, Institute of Molecular Biology and Genetics, 83031 Ariano Irpino, Italy

**Keywords:** chronic kidney disease, SGLT2i, vaptans, RASi, GFR

## Abstract

A major paradigm in nephrology states that the loss of filtration function over a long time is driven by a persistent hyperfiltration state of surviving nephrons. This hyperfiltration may derive from circulating immunological factors. However, some clue about the hemodynamic effects of these factors derives from the effects of so-called nephroprotective drugs. Thirty years after the introduction of Renin-Angiotensin-system inhibitors (RASi) into clinical practice, two new families of nephroprotective drugs have been identified: the sodium-glucose cotransporter 2 inhibitors (SGLT2i) and the vasopressin receptor antagonists (VRA). Even though the molecular targets of the three-drug classes are very different, they share the reduction in the glomerular filtration rate (GFR) at the beginning of the therapy, which is usually considered an adverse effect. Therefore, we hypothesize that acute GFR decline is a prerequisite to obtaining nephroprotection with all these drugs. In this study, we reanalyze evidence that RASi, SGLT2i, and VRA reduce the eGFR at the onset of therapy. Afterward, we evaluate whether the extent of eGFR reduction correlates with their long-term efficacy. The results suggest that the extent of initial eGFR decline predicts the nephroprotective efficacy in the long run. Therefore, we propose that RASi, SGLT2i, and VRA delay kidney disease progression by controlling maladaptive glomerular hyperfiltration resulting from circulating immunological factors. Further studies are needed to verify their combined effects.

## 1. Introduction

Before the end of the twentieth century, the nephrological community focused on a single state of kidney pathology, the kidney failure stage. The loss of kidney function, when dialysis was not an option, meant certain death for patients. Therefore, scientists devoted all their efforts to the problem of the management of patients who could not survive a kidney insult. With the invention of dialysis techniques, the primary concern for nephrologists remained on how to improve this technique and prolong survival. However, physicians were also paying little attention to the pre-dialysis period because they had no methods to slow down the kidney disease. In a minority of cases, such as immunological disorders, physicians had valuable treatments, namely steroids and immunosuppressive drugs. Otherwise, there was no nephroprotective drug available, and physicians could not slow down kidney function decline. Indeed, in this period, most of the literature widely used the term “chronic renal failure”, which appeared for the first time in the 1950s in a manuscript by Platt on plasma electrolytes [1]. “Failure” was the keyword, and it gained favors in the 1960–1970s, with a peak of publications in 2005 (783 papers). After 2005, the number of publications using the term “kidney failure” rapidly declined. This decline has not been an unconscious modification of the medical dictionary: it has been a deliberate decision that occurred in 2002, when the KDOQI commission decided to avoid the terms “renal” (because the Latin name is unfamiliar to the English-speaking population) in favor of “kidney” and to prevent the use of “failure” in favor of “disease”. The latter was due to a critical shift in attention of the medical community: the introduction of the angiotensin-converting enzyme inhibitors (ACEi) in the 1980s and, after that, of the angiotensin-II-receptor blockers (ARB) in 1991, collectively called renin, angiotensin system inhibitors (RASi), which changed the therapeutic landscape [2,3] and prolonged the kidney function in nephrological patients. Therefore, the medical community started to focus on the pre-dialysis period and decided to adopt an old term, “chronic kidney disease” (CKD), which appeared for the first time in 1934 in a pathology paper concerning the decrease in glomeruli [4]. Before 2002 this term was used seldom in the literature: from 1949 to 2002 it has been used only 126 times. The advent of RASi changed the view of the pre-dialysis period. Their use as first-line drugs for managing proteinuria and slowing kidney disease progression drove the attention towards the initial phases of kidney diseases [5]. The possibility to modify the natural history of kidney diseases induced a thoroughly cultural revolution, stemming from the change of the vocabulary such as: (i) the use of “chronic kidney disease” in place of “kidney failure” because now a treatment was available to slow down the disease; (ii) the introduction of a new method to estimate the kidney function, that is the MDRD equation (which was then followed up by other formulas), which was based on creatinine and did not require urine collection, thereby gaining great success among internists for its simple application and (iii) a new staging system arbitrarily divided in five stages (which required then further modification to account for the prognostic value of each phase) to provide further attention to the early phases of kidney disease (KDOQI classification system) [6].

**Figure 1:**
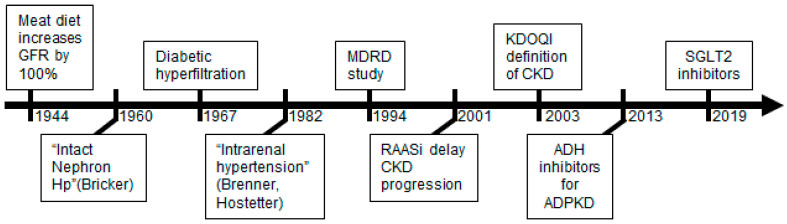
Timeline of the renal reserve concept and the discovery of nephroprotective drugs. The observation of an increase in glomerular filtration rate (GFR) after a meat meal and in diabetes led to the hypothesis of intrarenal hypertension. The MDRD study introduced a new equation to estimate GFR and showed it is not sufficient to avoid meat meals. With the advent of RASi it has been possible to slow down chronic kidney disease (CKD) and hence a new staging system for CKD was designed. Two additional drug classes were then found to slow down CKD.

It took about 30 years to discover two new drug classes exerting nephroprotective effects: the vasopressin receptor antagonists (VRA) or vaptans [7], and the sodium-glucose transporter 2 inhibitors (SGLT2i) [8] (Figure 1). 

The first vaptan to be discovered was OPC-21268 in 1991 [9] through a screening of a chemical library, followed by its chemical optimization which led to Mozavaptan (1992), Relcovaptan (1993), Satavaptan (1996), Conivaptan (1997), Lixivaptan (1998), and Tolvaptan (1998).

RASi, SGLT2i, and VRA are the only known nephroprotective drugs able to reduce renal disease progression. It is unclear why these drugs exert a similar nephroprotective action despite dramatic differences in their pharmacodynamics. Notably, it went unnoticed that all these drugs share the paradoxical reduction of the estimated glomerular filtration rate (eGFR) at the therapy’s onset. Therefore, the nephroprotective effect can be observed only after a long time (chronic treatment) and consists of a slower disease progression rather than an improvement of eGFR. Accordingly, nephroprotective effects are less remarkable at ending stages of chronic kidney disease (CKD) [10]. This paradoxical effect somehow resembles the counterintuitive use of beta-blockers for heart failure, as they too initially reduce cardiac output, whereas they are cardio-protective in the long run [11]. 

A way to interpret these phenomena is within the framework of the glomerular hyperfiltration theory. The hyperfiltration paradigm has its roots in the 1940s when Shannon et al. first observed an increase of GFR after a meat diet [12]. Subsequently, several authors confirmed that meat food, as well as other stimuli, can induce a functional, reversible increase of GFR, which was termed glomerular hyperfiltration. In 1967, Ditzel et al. also identified glomerular hyperfiltration in diabetic patients [13]. These data (and possibly the “intact nephron hypothesis” by Bricker [14]) motivated Hostetter and Brenner to formulate the theory of intrarenal hypertension in 1992 as a common ground for all kidney diseases [15,16]. The idea is that kidney diseases progress due to a constant hyperfiltration state, which was initially termed “intrarenal hypertension” [17]. As kidney damage progresses, the hyperfiltration in the remaining glomeruli maintains the GFR in a normal range. Indeed, clinicians and researchers can observe at this stage that the GFR is normal, but glomeruli are not able to further increase their filtration after a meat meal because they already work at the maximum level: this effect was called “reduced renal reserve” [18].

The great merit of this theory is that the reduction of glomerular hyperfiltration can explain the nephroprotective effect of RASi. In the following sections, we reanalyze evidence that RASi reduces eGFR and verify that this also applies to SGLT2i and VRA. Afterward, we will evaluate whether the reduction of GFR induced by these drugs correlates with their long-term nephroprotective efficacy. We relied on selected, well-known clinical trials on the issue.

## 2. Glomerular Hyperfiltration and Circulating Immunological Factors

Renal hypertrophy and glomerular hyperfiltration are mediated by several immunological factors and cells. 

Unfortunately, literature data regarding immunological factors have not been explored systematically. Therefore, we still lack a clear picture of the role of innate and adaptive immune systems on glomerular hyperfiltration.

This situation contrasts with the evidence that the immune system can deeply and reversibly change the GFR: (i) Interstitial nephritis, consisting of the infiltrate of immune cells (and often eosinophils) can impair GFR (ii) vasculitis and systemic lupus can induce a decrease of GFR (iii) antibody-mediated and complement-mediated glomerulonephritis (IgA, IgG, IgM, C3 nephropathies) can decrease the GFR. In all these forms, the GFR decrease is reversible, at least in the initial stages. However, most of the literature considers these effects due to histological damage and its subsequent repair. Even though this might be only part of the story, these diseases will not be reviewed here because the data are too scanty.

Cytokine storms (evoked in animal models by lipopolysaccharide LPS) and two chemokines, IL-1 and IL-6, are known to reduce the GFR, possibly though hemodynamic effects. Similarly, some chemokines such as TNF can increase the GFR. We will briefly review the evidence supporting the hemodynamical regulation of GFR by these immunological factors. Finally, we will shortly discuss whether these immune factors derive from innate or adaptive systems.

In kidney nephropathy, glomerular hyperfiltration is driven by TNFα [19], which modifies cell survival of glomerular mesangial and epithelial cells through nuclear factor-kB (NF-kB) signaling [20]. TNFα has a direct influence on glomerular hemodynamics [21]. Together with TNFβ, it might have a role in autophagy [22]. The inhibition of TNFα with monoclonal antibodies (e.g., infliximab) prevented renal function decline in the patients with rheumatoid arthritis [23]. Furthermore, a single dose of Infliximab appears to decrease eGFR as measured by cystatin C (but not with creatinine) [24]. At variance, two other TNFβ, adalimumab and etanercept, do not modify eGFR [25,26]. Interleukin(IL)-1β is another immune-mediated cytokine, with effects on glomerular hyperfiltration at least in the diabetic settings [27]. 

Interestingly, Anakinra, an anti- IL-1 β monoclonal antibody, decreases GFR measured by inulin method (https://www.ema.europa.eu/en/documents/variation-report/kineret-h-c-363-x-0042-epar-assessment-report-extension_en.pdf (accessed on 2 February 2022)). 

IL-6 is a pro-inflammatory cytokine implicated in several immunological diseases. It has been implicated in kidney disease progression in diabetes [28]. Accordingly, tocilizumab, an anti- IL-6 monoclonal antibody, can prevent loss of renal function in diabetic kidney disease [29]. T regulatory lymphocytes might also be involved in glomerular hyperfiltration: their depletion in db/db mice worsened glomerular hyperfiltration and albuminuria [30].

### The Role of Innate and Adaptive Systems

The immune system is divided into an innate immunity and an adaptive immunity. This distinction is very old and derives from the terms “acquired” and “natural” immunity in the 1950s and possibly before. 

In 1998, Rocket al. and Medzhitov et al. identified five human receptors for a protein similar to a protein studied in Drosophila and named Toll (the latter identified by the Nobel laureate Hoffman). Therefore they used the term “toll-like receptors” [31]. Subsequently, these receptors were found to be important for immune response, and became part of the “innate” immunity, being expressed on monocytes/macrophages and dendritic cells, natural killer cells, and endothelial cells.

At variance, the adaptive immune system is composed by cells able to modify their surface receptors, such as B- and T-lymphocytes.

In chronic degenerative diseases such as atherosclerosis, the innate immune system appears to play a major role [32].

Information regarding the role of innate and adaptive immune systems on the regulation of GFR is scant. 

Toll-like receptors are able to reduce intratubular urine flow rate [33]. These receptors are expressed on macrophages, which belong to the innate immune system. Accordingly, macrophages appear to be responsible for glomerular hyperfiltration, at least in specific mouse models of kidney disease [34]. Furthermore, chemokine levels deriving from the adaptive immune system do not induce glomerular hyperfiltration [35].

However, it should be emphasized that the subdivision between the innate and adaptive immune system is possibly quite artificial, and that the two systems are tightly linked by a network of cytokines [36].

Overall, we do not have sufficient data to clearly define the role of innate and adaptive systems on the glomerular hemodynamics. This parallels the extensive knowledge of the acute effects of many kidney diseases on the GFR, with very little data on the hemodynamic effects of cytokines. Therefore, future studies should try to address this problem.

This topic is timely, as most of the known immune-mediated effects on GFR in glomerulopathies are treated using RAS inhibitors, for reasons that are thoroughly discussed below. 

## 3. RASi and Initial eGFR Reduction 

The incidence of renal and cardiovascular events of long term RASi treatment was studied mainly in the ONTARGET trial (Ongoing Telmisartan Alone and in Combination with Ramipril Global Endpoint Trial) and in the TRANSCEND trial (Telmisartan Randomized Assessment Study in ACE Intolerant Participants with Cardiovascular Disease) [37]. In both trials, an initial temporary decrease in eGFR during treatment with RASi was observed. In contrast, the reduction of renal function over the years was slower in the treated group versus the placebo [38]. The effect on eGFR is transient since the studies confirmed the decrease observed at 2 weeks was not persistent after 8 weeks of treatment [39]. The analysis by Holtkamp et al. of the RENAAL (Reduction of Endpoints in Non-Insulin-Dependent Diabetes Mellitus with the Angiotensin II antagonist Losartan) trial highlighted that an initial significant decrease in eGFR was reported in the treated group versus placebo [39]. Notably, the authors noted that the initial decline in eGFR was inversely related to long-term protection. Therefore, the temporary decrease in eGFR was thought to reduce the glomerular hyperfiltration [40].

## 4. SGLT2i and eGFR Reduction

**Figure 2:**
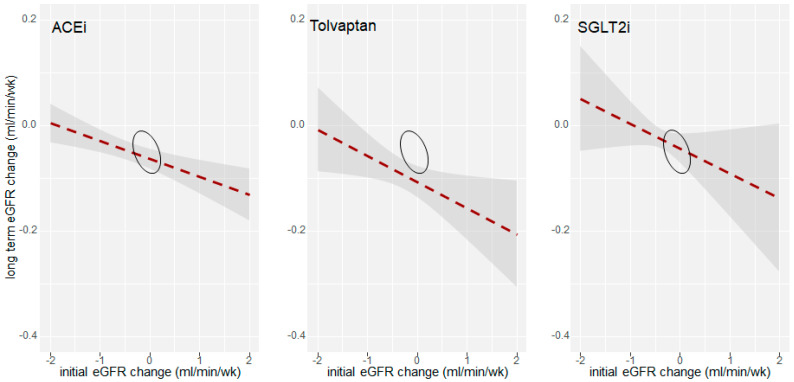
The extent of the initial drop in eGFR predicts the long term eGFR decline in patients treated with SGLT2i, Tolvaptan (a VRA), or ACEi. Negative values represent an eGFR loss over time. The horizontal axis reports the extent of acute fall in eGFR; the vertical axis reports the long-term effect on eGFR decline. Since these drugs are not reverting kidney damage, a long-term decrease of eGFR (negative values) should be expected, though at a lower rate than controls (values closer to zero). To allow comparisons among different trials, the eGFR loss has been divided by the weeks of observation. The more significant the initial drop in eGFR (horizontal axis), the greater the drug’s nephroprotective effect (vertical axis). ACEi data are elaborated from [39,40,50,51,52,53]. Tolvaptan data were elaborated from [7,10,54,55,56,57]. SGLT2i data were elaborated from [8,41,45,46,47,48,49]. Circles represent placebo effect.

A similar effect on eGFR has been recently observed for the SGLT2i. This new class of anti-diabetic drug blocks glucose reabsorption in the proximal tubule by SGLT2i. It has been approved to treat type II diabetes mellitus (T2DM). The EMPA-REG, CANVAS, and DECLARE trials [41,42,43] showed the beneficial renal effects of SGLT2i, supporting the hypothesis of SGLT2i nephroprotection. However, these trials were designed with renal outcomes as a secondary endpoint, and most subjects were at low risk of ESRD. Later, the CREDENCE trial was aimed to assess renal survival in a large population with DM2 and advanced renal disease and confirmed the nephroprotective effect of this new class of drugs [44]. Figure 2 summarizes the relation between change in eGFR and nephroprotection. Similarly to other dug classes discussed here, SGLT2i also reduce proteinuria, an effect consistent with a reduction of intraglomerular pressure [45,46,47,48,49].

## 5. VRA and eGFR Reduction 

Tolvaptan and conivaptan are the main VRA approved drug treatment for autosomal dominant polycystic kidney disease (ADPKD), aiming at slowing down the progression of kidney function loss [58]. The drug is a selective V2-receptor antagonist that blocks the hormone vasopressin, driven by increased second messenger cAMP in tubular cells [59]. The cAMP level has been related to cyst development and growth [60]. In summary, Tolvaptan down-regulates aquaporin-2 channels expression and reduces cAMP levels through modulation of the level of water and sodium, reduces the fluid intake in cysts and cystogenesis, and has been shown to slow down the kidney volume enlargement [7]. The most common adverse events are linked to its aquaretic effect (dehydration and sodium imbalances) and elevations of liver transaminases. The Tolvaptan efficacy and safety in ADPKD have been evaluated in TEMPO and REPRISE studies [7,59,61,62]. The experimental designs included ADPKD patients who had rapidly progressing disease but regular or moderately reduced kidney function (TEMPO3:4 study [7]) and subjects in advanced stages of the disease (CKD stages 3 and 4 initial) (REPRISE [61,62]). Tolvaptan is indicated in adults at risk of rapidly progressing ADPKD from CKD stage II-IV [63]. An initial and transitory decrease in kidney function was observed in patients with ADPKD treated with Tolvaptan [10]. Total kidney volume (TKV) has been qualified as a prognostic biomarker in patients with ADPKD [64] and used as the primary or secondary endpoint in clinical trials of renin-angiotensin blockade [43], Tolvaptan [54], and somatostatin analogs [65]. The Mayo Clinic’s current classification for ADPKD patients considers height-adjusted total kidney volume (HtTKV) and age as predictors of renal disease progression [66] to forecast eGFR decline over the years [67]. 

The analysis of available time-course data of eGFR in patients using Tolvaptan shows that the eGFR decline inversely relates with the acute eGFR decrease at Tolvaptan initiation. As shown in Figure 2, the extent of the initial drop in eGFR predicts the long-term GFR decline. Patients who had a greater reduction in eGFR levels during the first four weeks of Tolvaptan treatment had the most stable renal function during the years of observation. In addition, it is interesting to note that Tolvaptan also reduces albuminuria [39], which further suggests that its protective action onto the kidney is not limited to tubular functions, but on the glomerular hyperfiltration.

In previous publications, we have clearly shown that the absence of ADH in diabetes insipidus is accompanied by a persistent decrease of the GFR and by an absent hyper-filtrating response to a protein meal [18,68]. The same phenomenon is also present in nephrogenic diabetes insipidus. These data agree with the evidence that with the blockade of ADH receptor by tolvaptan, a decrease of GFR is present. However, as also suggested by an anonymous reviewer, at present it is difficult to talk about Tolvaptan in relation to hyperfiltration and further studies should confirm or reject the argument that Tolvaptan suppressed hyperfiltration (at least in part) by decreasing eGFR in the early stages of treatment.

### Immunological Factors in Nephroprotective Effects of RASi, SGLT2i, and VRA

Although RASi and VRA (and possibly SGLT2i) have a clear direct effect on glomerular hemodynamic, a more indirect effect mediated by immunological factors cannot be excluded.

RASi also have modulator effects on inflammation, contributing to organ protection. This may be related to the activation of inflammatory response by angiotensin type 1 receptors (AT1R). Furthermore, they may potentially modulate responses of Th17 and Treg lymphocytes. Furthermore, they also regulate Klotho and other anti-inflammatory factors [69].

SGLT2 inhibitors are known to modulate the inflammasome [70] and macrophage polarization [71].

Similarly, Tolvaptan has some effect on T-cells, although this has been correlated with the adverse effect of the drug onto the liver [72].

## 6. Other Examples

One major question from the previous discussion is whether all drugs that reduce the eGFR can be protective in the long run. This discussion will not take into account drugs known to reduce eGFR through nephrotoxic mechanisms such as inflammatory damage, acute interstitial nephritis, or glomerulonephritis (such as acetaminophen, lithium, acyclovir, beta lactams, vancomycin, cisplatin, methotrexate, thiazides, omeprazole, allopurinol) [73]. Therefore, we will focus on major examples of drugs reducing the eGFR through intrarenal hemodynamic changes: (i) aldosterone antagonists, (ii) non-steroidal anti-inflammatory drugs (NSAIDs), (iii) calcineurin inhibitors (CNI, e.g., cyclosporine/tacrolimus), and (iv) diuretics.

In an interesting overview proposed by Sato [49], RAS agents act on the efferent arteries. Conversely, NSAIDS (which are not nephroprotective), by inhibiting the Cyclo-oxygenase-2, impair afferent artery dilation without effects on the efferent artery of the glomeruli. 

It is, therefore, tempting to speculate that only drugs that reduce the eGFR by dilating the efferent arteriole are nephroprotective. This class of drugs comprise not only RAS, but also and mineralocorticoid receptor antagonists (MRA), endothelin-1, thromboxane A2, and reactive oxygen species. Indeed, MRA such as eplerenone and spironolactone reduce the eGFR in the acute phase; however, they also exert a nephroprotective effect on the long-run [74]. This has also been demonstrated for finerenone [75,76,77,78].

In agreement with the hypothesis carried out in the present paper, the extent of the long-term renal protective effect is proportional to the initial decrease of eGFR when MRA are used [49]. Endothelin receptor antagonists are also known to reduce eGFR; however this potentially nephroprotective effect is counterbalanced by the adverse events which make this approach unfeasible [79]. 

Thiazide diuretics (particularly chlorthalidone) appear also to exert anti-proteinuric [75] and nephro-protective actions [80]. It also reversibly reduces GFR [80] as the other classes of drugs reviewed here.

Furosemide also appears to reduce the GFR if diastolic dysfunction is present [81]. However, this effect depends on volume reduction, because furosemide has very few effects on GFR in animal models when volume status is controlled [82]. Consistently, furosemide has not been reported to reduce proteinuria.

Conversely, following the above hypothesis, the agents that vasoconstrict the afferent arteriole reduce the eGFR but have no nephroprotective effect. These agents comprise the NSAIDs, which are known to have no nephroprotective effect. Indeed, NSAIDs can interfere with the kidneys’ ability to autoregulate glomerular pressure and decrease GFR. However, at variance from the previously described agents, NSAIDs do not exert nephroprotective effects on the long-run and are associated with eGFR decline [83]. In addition to NSAIDs, the inhibitors of Nitric oxide (NO), prostanoids, kallikrein/kinin, and Atrial Natriuretic Peptide (ANP) would also be predicted to reduce eGFR acutely. Data regarding these approaches are sparse and will not be analyzed here.

Other drugs, such as calcineurin inhibitors (e.g., cyclosporine, tacrolimus), cause dose-dependent vasoconstriction of the afferent arterioles, leading to a decrease of eGFR. As expected, in the long run these drugs do not have nephroprotective effects. 

Finally, calcium channel blockers also elicit vasodilation on both the afferent and efferent arteriole. This double change leads to no difference in eGFR and no nephroprotective effect in the long-run [84]. Aliskiren, a rennin inhibitor, also has no known nephroprotective effects.

## 7. Discussion and Conclusions

Chronic kidney diseases are progressive medical conditions and an economic burden in ageing populations. Immune mechanisms and mechanic factors (e.g., hyperfiltration state and “intrarenal hypertension”) are involved in the progression of kidney disease and their interaction may define some previously unexplained kidney diseases.

In this review we reanalyze the acute effect of RASi, SGLT2i, and VRA on eGFR and we evaluated whether the reduction of GFR induced by these drugs correlates with their long-term nephroprotective efficacy. 

A recent review from Ellison [85] elucidated the linkage between SGLT2i and reduction of glomerular hyperfiltration in diabetic nephropathy, and perhaps in other diseases. 

However, several reports in literature highlighted the role of the immune system in the pathogenesis of renal hypertrophy and glomerular hyperfiltration. Data show that tumor necrosis factor (TNF) is elevated in the early stages of diabetic nephropathy. Therefore, it is possible that TNF is directly involved in the development of renal hypertrophy and its production may be mediated by sodium retention. In addition, it has been demonstrated that TNF can affect the growth of mesangial cells under experimental conditions.

Data shown in Figure 2 suggest that the extent of acute eGFR fall after initiation of nephroprotective agents (SGLT2i, RASi, or VRA) is directly correlated with renal survival. A previous report shows that Tolvaptan slows down CKD progression and reduces the rate of patients who need to undergo kidney replacement therapy [55]. Indeed, Edwards et al. [55] showed that the eGFR decline is stable over time in Tolvaptan treated patients between 1 and 5 years and in those treated for more than five years across different CKD stages. 

We previously discussed vasopressin’s potential role in controlling glomerular hemodynamics, allowing the GFR increase in acute conditions. We demonstrated that the absence of vasopressin (AVP) in diabetes insipidus lowers the basal GFR and prevents the glomerular hyperfiltration induced by a meat meal [68]. Therefore, the reduction of eGFR after Tolvaptan (which mimics a diabetes insipidus) entirely agrees with this previous observation.

**Figure 3:**
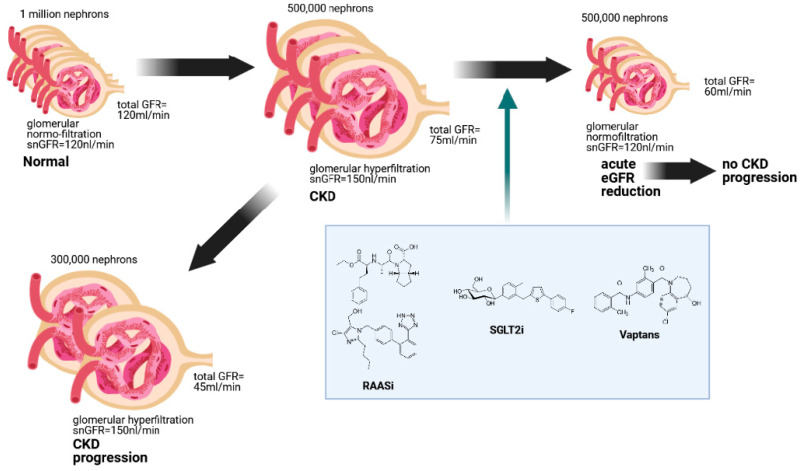
Hypothesis on the shared nephroprotective property of RASi, SGLT2i, and VRA.

We suggest that the more prominent reduction in eGFR at the beginning of treatment corresponds to a better outcome over the years in terms of the rate of progression of kidney disease. It has been described that Tolvaptan exerts an acute hemodynamic effect with a transitory reduction in eGFR and a small increase in creatinine levels [10] after treatment initiation. This effect is reported to be most effective during the first months of treatment [54]. However, this transitory reduction seems to revert after Tolvaptan suspension [57]. Therefore, as shown in Figure 3, we propose that RASi, SGLT2i, and VRA delay kidney disease progression by taking control over a maladaptive glomerular hyperfiltration. Further studies are needed to verify their combined effects.

## 8. Future Perspectives

The main consequence of this view is that VRA and SGLT2 inhibitors might exert nephroprotective actions well outside their main indications for ADPKD and diabetic nephropathy, respectively. Furthermore, it is possible that they have additive nephroprotective actions, and therefore their combination might be even more efficacious when combined with RASi. Somehow, this hypothesis has now been advanced for SGLT2 inhibitors, which appear to have protective effects on the cardiovascular outcomes even in the absence of diabetes. Whether this also applies to VRA has not been addressed yet in the literature.
